# The enteric nervous system deficits in autism spectrum disorder

**DOI:** 10.3389/fnins.2023.1101071

**Published:** 2023-08-24

**Authors:** Xinnian Wang, Ruijin Tang, Zhen Wei, Yang Zhan, Jianping Lu, Zhiling Li

**Affiliations:** ^1^CAS Key Laboratory of Brain Connectome and Manipulation, The Brain Cognition and Brain Disease Institute, Shenzhen Institute of Advanced Technology, Chinese Academy of Sciences, Shenzhen, China; ^2^School of Life Science, USTC Life Sciences and Medicine, Hefei, China; ^3^Department of Child Psychiatry and Rehabilitation, Affiliated Shenzhen Maternity and Child Healthcare Hospital, Southern Medical University, Shenzhen, China; ^4^Department of Child and Adolescent Psychiatry, Shenzhen Kangning Hospital, Shenzhen Mental Health Center, Shenzhen, China

**Keywords:** autism spectrum disorder, enteric nervous system, gut motility, genetical factor, environmental factor

## Abstract

Gastrointestinal (GI) disorders are common comorbidities in individuals with autism spectrum disorder (ASD), and abnormalities in these issues have been found to be closely related to the severity of core behavioral deficits in autism. The enteric nervous system (ENS) plays a crucial role in regulating various aspects of gut functions, including gastrointestinal motility. Dysfunctional wiring in the ENS not only results in various gastrointestinal issues, but also correlates with an increasing number of central nervous system (CNS) disorders, such as ASD. However, it remains unclear whether the gastrointestinal dysfunctions are a consequence of ASD or if they directly contribute to its pathogenesis. This review focuses on the deficits in the ENS associated with ASD, and highlights several high-risk genes for ASD, which are expressed widely in the gut and implicated in gastrointestinal dysfunction among both animal models and human patients with ASD. Furthermore, we provide a brief overview of environmental factors associated with gastrointestinal tract in individuals with autism. This could offer fresh perspectives on our understanding of ASD.

## Introduction

Autism spectrum disorder (ASD) is a prevalent neurodevelopmental condition characterized by difficulties in communication and social interaction, as well as repetitive behaviors or interests (Kanner, 1968; Volkmar et al., 2014). It is a growing public health concern, particularly in children, with an estimated prevalence of 1:59 among children and 1:100 among adults.

Apart from the core symptoms, ASD patients also experience various comorbidities, particularly gastrointestinal (GI) symptoms (Matson and Goldin, 2013). Research has indicated that individuals with autism frequently experience elevated occurrences of diarrhea, abdominal discomfort, and constipation in comparison to the general population (Horvath et al., 1999; Wasilewska and Klukowski, 2015). GI dysfunction not only affects other complications of ASD but also has a unique relevance to cognitive deficits in autistic individuals. Patients with GI disorders tend to experience more severe social withdrawal and other maladaptive behaviors, such as irritability, are frequently observed in ASD patients with GI issues (Chaidez et al., 2014; Madra et al., 2020). The aforementioned findings suggest that GI dysfunction may have a significant impact on the development of behavioral abnormalities in individuals with ASD, at least in certain subgroups of this population (Table 1). However, it remains unclear whether the gastrointestinal dysfunctions are a consequence of ASD or if they directly contribute to its pathogenesis. This review focuses on the deficits in the ENS associated with ASD, and highlights several high-risk genes for ASD, which are expressed widely in the gut and implicated in gastrointestinal dysfunction among both animal models and human patients with ASD. Furthermore, we provide a brief overview of environmental factors associated with gastrointestinal tract in individuals with autism. This could offer fresh perspectives on our understanding of ASD.

**Table 1:** Susceptibility genes relevance to GI dysfunction in ASD.

	References	Genes	Molecular phenotypes	ASD symptoms	GI dysfunction	Samples
Synaptic dysfunction mutation genes	Soorya et al. (2013)	*Shank3*	*#*	Impaired social interaction, evasive eye contact	Gastroesophageal reflux, constipation or diarrhea	PMS patients
	James et al. (2019)	*Shank3*	Reduced serotonin-positive EECs	*#*	Reduced DT peristalsis frequency, and prolonged DT transit time	*Shank3* KO zebrafish
	Sauer et al. (2019)	*Shank3*	Decreased *Shank3* gene expression in gut epithelial cells compared to wild type controls	*#*	#	*Shank3* KO mice
	Hosie et al. (2019)	*Nlgn3*	Increased Hu and nNOS myenteric neurons in the small intestine; Unchanged colonic GABAergic neurons	ASD behavior	Diarrhea and chronic gut pain; faster small intestinal transit and unchanged colonic motility	ASD patients; *NL3^R451C^* mice
	Leembruggen et al. (2020)	*Nlgn3*	Unchanged nNOS or calretinin motor neurons	*#*	Faster colonic motility	*Nlgn3^−/−^* mice
	Robinson et al. (2023)	*Caspr2*	Unchanged organization of neurons within the myenteric plexus; *Caspr2* is expressed in enteric sensory neurons in both the small intestine and colon	*#*	No significant difference in whole GI transit time, altered colonic contractions and accelerated colonic motility	*Caspr2−/−* mice
Signaling molecule dysfunction-related genes	Marler et al. (2015)	*SLC6A4*	Elevated whole-blood serotonin	Alterations in brain, behavior	Lower GI symptoms and constipation	82 children and adolescents with ASD
	Margolis et al. (2016)	*SLC6A4*	Decreased enteric neurons, increased 5-HT clearance	ASD-like behavior	Slow GI transit and diminished peristaltic reflex activity	SERT Ala56 mice
	Bernier et al. (2014)	*CHD8*	50% reduction of enteric neurons	ASD behavior, increased head size	GI disturbance, such as constipation and diarrhea; impairment of GI motility	ASD patients with CHD8 mutations; Zebrafish with CHD8 mutations
Other high-susceptible ASD genes	Whalen et al. (2012)	*Tcf4*	*#*	Stereotypic movements, including arm flapping, hand biting, movement of fingers and wrists	Constipation and gastroesophageal reflux	PTHS patients
	Grubišić et al. (2015)	*Tcf4*	#	*#*	Reduced gut transit velocities *in vivo*	TCF4 functional deletion mice
	Siper et al., 2017	*Foxp1*	*#*	Delays in early motor and language milestones, language impairment,	Constipation	Children and adolescents with mutations in FOXP1
	Frohlich et al. (2019)	*Foxp1*	#	*#*	Impaired colonic contractility and prolonged total GI transit	*Foxp1^+/−^* mice
	Campbell et al. (2009)	*c-Met*	Over-representation of the *Met* rs1858830 C allele in ASD individuals with GI dysfunction	Alterations in brain, behavior	Gastrointestinal conditions	ASD individuals with a gastrointestinal condition report
	Russo et al. (2009)	*c-Met*	Decreased HGF levels in the ASD individuals with GI dysfunction	Alterations in brain and behavior	Chronic digestive disease, most with ileo-colonic lymphoid nodular hyperplasia	Serums from autistic children with GI dysfunction

# means not mentioned in the reference.

## Enteric nervous system

The gastrointestinal tract serves as the exclusive pathway for nutrient absorption, and it plays a crucial role in breaking down food into absorbable nutrients while eliminating waste. Additionally, the GI tract has the ability to defend itself against toxins, physical damage, irritants, and various bacteria that inhabit its length. Fortunately, these complex and essential functions are primarily regulated by the enteric nervous system (ENS), which is a local nervous system (Furness, 2012).

**Figure 1:**
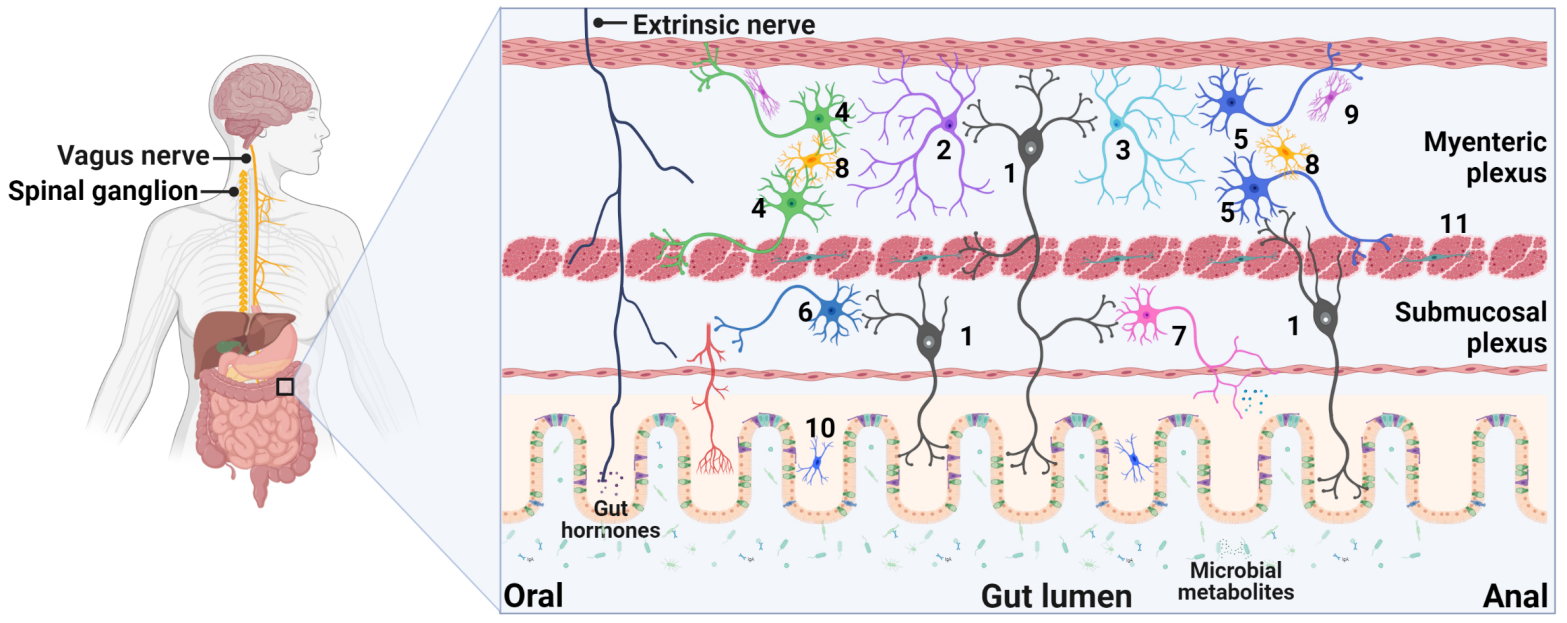
Schematic representation of the enteric nervous system (ENS). The ENS comprises a sophisticated network of neurons and glia organized into two ganglionated plexuses in mammals. The submucosal plexus is situated between the muscularis mucosa and circular muscle, while the outer myenteric plexus is located between the circular and longitudinal muscle layers. Herein, we present an enumeration of the principal cell types within the enteric system: 1, Myenteric/submucosal intrinsic primary afferent neuron; 2, Ascending interneuron; 3, Descending interneuron; 4, Excitatory motor neuron for circular/longitudinal muscles; 5, Inhibitory motor neuron for circular/longitudinal muscles; 6, Vasodilator neuron; 7, Secretomotor neuron; 8, Protoplasmic type I glia; 9, Fibrous type II glia; 10, Subepithelial type III glia; 11, Intramuscular type IV glia. Additionally, the ENS is also innervated by extrinsic nerves, including the vagal and spinal nerves. This figure was created with BioRender.com, with permission.

The ENS is a vital component of the autonomic nervous system that spans the entire length of the gastrointestinal tract. It consists of a complex network of neurons and glia organized into two ganglionated plexuses in mammals, namely, the submucosal (Meissner’s) plexus located between the muscularis mucosa and circular muscle as well as the outer myenteric (Auerbach’s) plexus situated between the circular and longitudinal muscle (Figure 1). Enteric neurons comprise a diverse population of cells, encompassing intrinsic sensory neurons, ascending and descending interneurons, and motor neurons. The myenteric plexus predominantly houses the neurons that govern intestinal contractile patterns and the circuits that synchronize these patterns with other intestinal behaviors. Meanwhile, the submucosal plexus primarily contains neurons that project to the mucosa and are involved in regulating secretion, vasodilation, and absorption (Furness, 2006). Enteric glia cells (EGCs), the non-neuronal components of the ENS, display diverse morphologies and are situated in distinct microenvironments depending on their location within enteric ganglia, along fiber tracts, or dispersed throughout the smooth muscular or mucosal layers of the gut (Boesmans et al., 2015). Initially regarded as supportive cells surrounding enteric neurons and nerve fibers, emerging research has challenged the traditional characterization of EGCs and demonstrated their involvement in regulating broader gut physiology through interactions with other cell types such as epithelial and immune cells. EGCs modulate synaptic signaling, regulate gut motility patterns, and help maintain intestinal barrier integrity (Seguella and Gulbransen, 2021).

**Figure 2:**
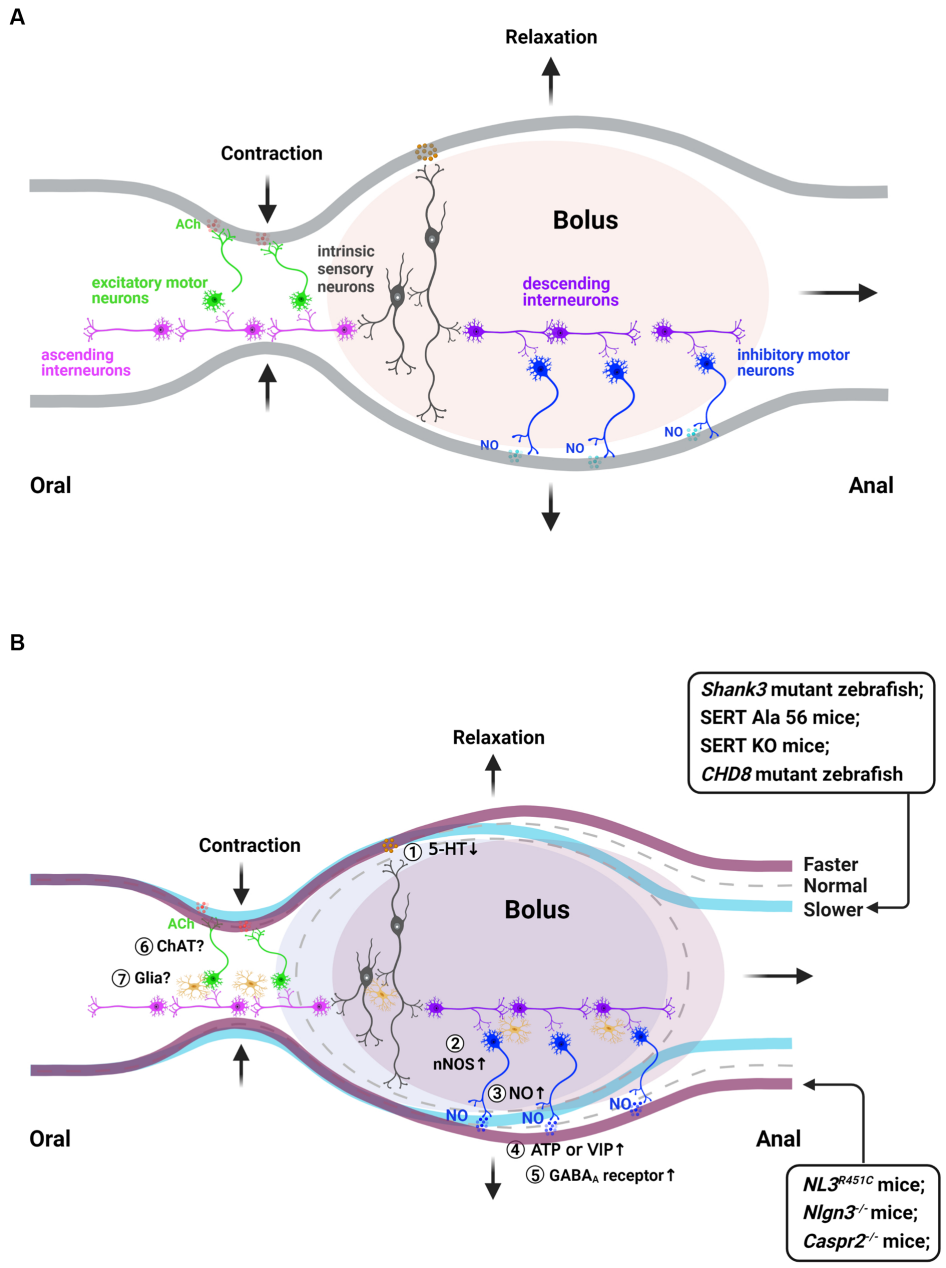
Schematics illustrating the fundamental circuitry regulating gut contraction and relaxation in normal condition, as well as possible mechanisms for gastrointestinal dysmotility in animal models with certain ASD—associated gene mutation. **(A)** The essential neuronal network responsible for coordinating peristalsis, a classic gastrointestinal motility pattern, comprises a five-neuron unit featuring an intrinsic sensory or primary afferent neuron (IPAN) that detects mechanical or chemical stimuli from the intestinal lumen. Subsequently, this IPAN activates excitatory or inhibitory motor neurons through ascending or descending interneurons. Consequently, an oral contraction of the intestinal muscle and distal relaxation occur in response to the stimulus. Additionally, enteric glia actively participate in regulating intestinal motility. **(B)** Potential mechanisms underlying gastrointestinal dysmotility in animal models with specific gene mutations associated with ASD. They include: (i) serotonergic signaling pathway: a reduction in serotonin-positive enteroendocrine cells, as well as alterations in SERT activity, may potentially impact the 5-HT signaling pathway that regulates enteric neuronal development and gastrointestinal motility ①; (ii) inhibitory pathway: aberrant innervation of GI smooth muscle by nNOS neurons, or an increase in the number of nNOS+ neurons or varicosities may lead to elevated levels of NO in the ENS, potentially contributing to gastrointestinal dysmotility in animal models of autism ②③④; (iii) GABA receptor pathway: activation of GABA and GABA_A_ receptors may potentially contribute to gastrointestinal motility, including the modulation of sensitivity toward GABA_A_ receptor modulators, such as alterations in the abundance and/or composition of GABA_A_ receptors ⑤. Additionally, (iv) Alterations in glia activity and an imbalance in the ratio between excitatory and inhibitory neurons within the enteric nervous system may contribute to gastrointestinal dysmotility observed in animal models of autism ⑥⑦. Ach, acetylcholine; ATP, adenosine triphosphate; ChAt, Choline acetyltransferase; GABA_A_, γ-aminobutyric acid A; NO, nitric oxide; nNOS, neuronal nitric oxide synthase; VIP, vasoactive intestinal peptide; 5-HT, serotonin. This figure was created with BioRender.com, with permission.

To maintain whole body homeostasis, the GI tract generates various patterns of motility, including peristaltic, accommodating, mixing and segmenting activities that not only vary according to different regions along the gut but also in response to dietary status (Sanders, 2008). Accumulating evidence has established a consensus that the ENS serves as the primary initiator of intricate motility patterns (Huizinga et al., 2011; Schneider et al., 2019). To “decide” what to do, intrinsic sensory neurons within the ENS respond to the mechanical or chemical stimulation of the intestinal wall. These signals are then transmitted to interneurons that project in both oral (ascending) and anal (descending) directions, ultimately contacting excitatory and inhibitory motor neurons, respectively (Figure 2). The enteric motor neurons activate the contraction and relaxation of the circular and longitudinal muscles through various layers of interstitial cells of Cajal (ICCs), platelet-derived growth factor receptor (PDGFR)(+) cells, enteric glial cells, and intestinal macrophages (Huizinga and Lammers, 2009; Sanders et al., 2010, 2016).

With the advancement of genetically encoded tools, we have gained a profound comprehension of integrated physiology in the GI tract. Recently, it has been elucidated that distinct motor patterns arise from structural variations in the functional organization of enteric circuits (Li et al., 2019). Certain genes, such as *Sox6* and *Celsr3*, are implicated in the organization of enteric neuronal projections that contribute to gastrointestinal motility (Sasselli et al., 2013; Memic et al., 2018).

GI motor disorders frequently co-occur in some individuals with ASD, suggesting the presence of ENS dysfunction. In the following section, we will provide a summary of dysmotility observed in various animal models of ASD induced by gene mutations.

## Genetic risk factors relevance to GI comorbidities in ASD

Although genetic risk is a major contributor to idiopathic ASD, the specific genetic alterations underlying most cases have yet to be identified. Many ASD-associated genes encode proteins involved in synapse formation and maintenance. Some of these mutations in synaptic genes have also been found to be expressed in the ENS, such as *Shank3* and neuroligin 3 (Huett et al., 2009; Raab et al., 2010; Ellis et al., 2012; Zhang et al., 2013; Niesler and Rappold, 2021). Additionally, *SLC6A4* and *CHD8*, involved in specific signaling pathways, also play a crucial role in the development of the ENS (Wade et al., 1996; Bernier et al., 2014). Here, we summarize findings from studies utilizing these genetic models and patient data.

*Shank3*, a well-studied member of the *Shank* gene family, is located on mouse chromosome 15E3 (human location: 22q13.3) and encodes a scaffolding protein in the postsynaptic density that is involved in clustering or assembling receptors, adhesion molecules, and signaling molecules (Jiang and Ehlers, 2013). Haploinsufficiency of the *Shank3* gene causes Phelan-McDermid syndrome (PMS), which frequently manifests with autistic or autistic-like behavior among patients (Phelan and McDermid, 2012; Sarasua et al., 2014). Moreover, PMS patients have reported experiencing gastrointestinal distress such as gastroesophageal reflux, cyclical vomiting, diarrhea, and/or constipation (Phelan and McDermid, 2012; Soorya et al., 2013), indicating a correlation between *Shank3* mutations and GI functions. In terms of animal models, significant progress has been made in investigating the biological mechanisms underlying gastrointestinal symptoms. For instance, a zebrafish *Shank3* mutant model of autism showed that mutations in *Shank3* resulted in abnormalities of GI transit and motility due to reductions in serotonin-positive EECs involved in subsequent secretion and peristalsis-associated neural pathways (James et al., 2019). Additionally, *Shank3* is expressed in the GI epithelium, and altered GI morphology and microbiota composition, along with elevated levels of zonulin-1 and IL-6, have been observed in *Shank3*-deficient mice, suggesting that extracerebral factors contribute to the phenotype observed in *Shank3*-deficient mice (Sauer et al., 2019).

Neuroligin, a postsynaptic cell-adhesion molecule, interacts with presynaptic and intracellular partners, such as neurexins and postsynaptic scaffolding proteins, to facilitate synaptic maturation and transmission (Krueger et al., 2012). Patients with ASD have been found to carry mutations in neuroligin-3 (*Nlgn3*), resulting in an arginine-to-cysteine substitution at the 451st amino acid residue (R451C; Jamain et al., 2003; Hosie et al., 2019; Zhang et al., 2022). GI-related problems, such as diarrhea and chronic gut pain, have also been confirmed in patients with the NL3^R451C^ genetic mutation (Hosie et al., 2019). To investigate potential mechanisms underlying these disorders, two mouse models of autism associated with NLGN3 were utilized: NL3^R451C^ knock-in mice and *Nlgn3* KO mice. Both mouse models exhibit gut dysfunction, characterized by faster *in vivo* small intestinal motility, heightened sensitivity to GABA_A_ receptor modulation in colonic enteric neurons, altered neuronal numbers in the small intestine for NL3^R451C^ mice (Hosie et al., 2019), as well as faster colonic motility and enlarged colonic diameter in *Nlgn3* KO mice (Leembruggen et al., 2020). This suggests that NLGN3 exerts a modulatory effect on neural pathways within the ENS, potentially contributing to the pathophysiology of GI disorders in ASD patients with NLGN3 mutations.

*Caspr2* (known as CNTNAP2), a member of the Neurexin family that acts as a synaptic cell-adhesion molecule involved in forming trans-synaptic complexes, was the first gene discovered to have rare and common mutations that increase susceptibility to ASD (Alarcon et al., 2008; Penagarikano and Geschwind, 2012). It has been reported that variations in or the absence of *Caspr2* have been associated with a range of symptoms related ASD in both human patients and animal models, such as language regression, paresthesia, social impairment (Strauss et al., 2006; Peñagarikano et al., 2011; Penagarikano and Geschwind, 2012). Recently, Robinson’s research revealed that apart from the already known brain regions, *Caspr2* is also expressed in enteric sensory neurons of adult mice (Robinson et al., 2023) and lack of *Caspr2* alters GI motility suggesting enteric sensory dysfunction may contribute to ASD-related GI symptoms (Robinson et al., 2023).

*SLC6A4,* encoding the serotonin (5-HT) reuptake transporter (SERT), together with 5-HT, has been demonstrated to play crucial roles in both brain and gut development as well as long-term function (Li et al., 2011; Gershon, 2013; Homberg et al., 2013). Rare mutations in multiple alleles of *SLC6A4* that cause hyperserotonemia have been identified as ASD risk factors (Sutcliffe et al., 2005; Veenstra-VanderWeele et al., 2012). Additionally, elevated levels of whole blood serotonin have been consistently observed as a biomarker in approximately 30% of children diagnosed with ASD (Schain and Freedman, 1961; Gabriele et al., 2014; Muller et al., 2016). It has been reported that whole blood serotonin levels are associated with lower GI symptoms, such as abdominal pain, stool retention, and large bowel movements in children and adolescents with ASD. However, functional constipation—the most frequent complaint—is not associated (Marler et al., 2015). As observed in human patients, a knock-in mouse model overexpressing the most common SERT variant (Gly56Ala) in ASD children not only exhibits ASD-like behaviors but also displays abnormalities in the ENS (Margolis et al., 2016). The Gly56Ala mice exhibited ENS hypoplasia, slow gastrointestinal transit, reduced peristaltic reflex activity, and increased proliferation of crypt epithelial cells (Margolis et al., 2016). Alterations in SERT-mediated 5-HT clearance could impact all ENS functions mediated by 5-HT, thus investigating the role of 5-HT and SERT in brain-gut disorders may offer valuable insights into ASD treatment.

*CHD8,* located on 14q11.2, is a critical player in various processes such as transcriptional regulation, epigenetic remodeling, cell proliferation promotion and RNA synthesis regulation through encoding chromodomain helicase DNA-binding protein 8 (Thompson et al., 2008; Nishiyama et al., 2012). In 2012, allelic variants of this gene were initially associated with autism (O'Roak et al., 2012a,b); Subsequently, it was identified as one of the few genes recurrently disrupted in the condition. Individuals with a deleterious *CHD8* mutation typically exhibit autism marked by macrocephaly, distinctive facial features, and digestive issues (Bernier et al., 2014). It has been reported in a previous review that children with *CHD8* mutations exhibit a significantly higher incidence of constipation compared to those without the mutation (60%–26%; Li and Zhou, 2016). Regarding the animal data, disruption of *CHD8* in zebrafish reproduces human phenotypic features, such as macrocephaly resulting from expansion of the forebrain/midbrain and impaired gastrointestinal motility due to reduced colonization by postmitotic enteric neurons in the GI tract (Bernier et al., 2014).

Furthermore, other ASD risk genes such as transcription factor 4 (*Tcf4*), Fork-head box protein P1 (*Foxp1*), and *c-Met* have also been implicated in the GI phenotypes of ASD; however, their expression in the ENS has not yet been determined (Campbell et al., 2009; Russo et al., 2009; Horn et al., 2010; Whalen et al., 2012; Peng et al., 2013; Grubišić et al., 2015; Meerschaut et al., 2017; Siper et al., 2017; Frohlich et al., 2019). Mutations in these genes were identified in both patients and mice exhibiting a spectrum of gastrointestinal symptoms, including constipation and gastroesophageal reflux disease.

Taken together, the presence of GI abnormalities in autism suggests a potential link between GI dysfunction and the pathophysiology of autism. Further prospective studies on specific subgroups of individuals with ASD are necessary to determine the prevalence of gastrointestinal complications. These genetic studies may lead to earlier diagnoses, interventions, and improved treatment for ASD in the future.

## Environmental risk factors

In addition to the aforementioned genetic factors, epidemiological studies have indicated that environmental risks also play a crucial role in the development of ASD (Bölte et al., 2018). Here we summarize several biological environmental risk factors: prenatal influences, gut microbiota, and zinc, which alter intestinal characteristics in individuals with autism.

### Prenatal/perinatal factors in ASD

Emerging evidence suggests that prenatal factors play a pivotal role in shaping the composition of gut microbiota. The ASD mouse model, induced by maternal immune activation (MIA), exhibits dysbiosis of gut microbiota resembling the clinical features observed in ASD patients (Hsiao et al., 2013; Lim et al., 2017; Tartaglione et al., 2022). Moreover, pre-conceptional microbiota transplantation has the potential to ameliorate MIA-induced neurodevelopmental abnormalities by inhibiting interleukin-17a signaling during gestation (Lammert et al., 2018). The fisssswndings suggest that modulation of the maternal immune response may serve as a potential therapeutic strategy for autism. However, there is limited research investigating the association between prenatal or perinatal factors and gastrointestinal problems involving the ENS in individuals with autism.

### Gut microbiota in ASD

The gut microbiota is a complex ecosystem that resides in the intestinal tract, consisting of approximately 100 trillion microorganisms, with two bacterial divisions—*Bacteroides* (~20%) and *Firmicutes* (~70%)—accounting for 90% of its composition (Eckburg et al., 2005; Qin et al., 2010). In addition to maintaining intestinal homeostasis, the microbiota has been implicated in regulating brain physiology and behavior (Sherwin et al., 2019).

It is well-known that individuals with ASDs often exhibit imbalances in the gut microbiota compared to neurotypical controls, which manifests as alterations in bacterial diversity and a reduction in the abundance of beneficial bacteria (Strati et al., 2017; Xu et al., 2019; Sivamaruthi et al., 2020). Analysis of the fecal microbiota in children with ASDs reveals an overgrowth of pathogenic strains and a significant reduction in the Bacteroidetes/Firmicutes ratio (Adams et al., 2011; Strati et al., 2017; Settanni et al., 2021). For instance, elevated levels of *Clostridium* and *Desulfovibrio* were frequently observed in ASD individuals and were found to be correlated with the severity of autism symptoms as well as various gastrointestinal disorders (Song et al., 2004; Parracho et al., 2005). Moreover, alterations in microbial metabolites, such as short-chain fatty acids (SCFAs), were also discovered to be associated with the severity of ASD (Adams et al., 2011; Wang et al., 2012). Acetate, propionate, and butyrate are all SCFAs produced through the anaerobic fermentation of indigestible carbohydrates. Taken together, these studies indicate that certain types of ASDs may exhibit alterations in the composition and function of their gut microbiota, highlighting the potential therapeutic approach of manipulating the gut microbiota for ASD. This primarily involves the use of probiotics, fecal microbiota transplantation, and microbiota transfer therapy (Cryan et al., 2019). Although modifying the composition of gut microbiota can improve behaviors and gastrointestinal symptoms in individuals with ASD or animals, the mechanistic understanding of interactions between microbes and the nervous system remains elusive. Because no specific microbial species has consistently changed across all ASD-related studies so far, which is why controversies always exist.

The ENS is exposed to and engages with the gut microbiota. While the cellular architecture of the ENS is largely established at birth, the process of functional maturation in enteric wiring occurs within the postnatal gut microenvironment under the influence of the gut microbiota (Obata and Pachnis, 2016). Given its crucial role as a key constituent of the microbiota-gut-brain axis, an extensive investigation into the involvement of the ENS in ASD models is warranted. Moreover, it is imperative to consider other variables such as the size of the sample, dietary patterns, and regional disparities that can all contribute to the heterogeneity of gut microbiota (Sivamaruthi et al., 2020). This area of research is still expanding, thus necessitating further investigations with substantial sample sizes of both autistic and neurotypical children who have not undergone antibiotic treatment to identify the precise role of gut microbiota dysbiosis in the development of ASD.

### ENS and zinc deficiency in ASD

Zinc, one of the most prominent trace metals, plays a vital role in the development of newborns by regulating neurogenesis, neuronal migration and differentiation, which are essential for shaping cognitive development and maintaining healthy brain function (Zoroddu et al., 2019). However, the prevalence of zinc deficiency has been found to be significantly higher among individuals with ASD compared to age-matched healthy controls (Grabrucker, 2013; Pfaender and Grabrucker, 2014).

Additionally, Zinc is essential for the proper development and maintenance of the gastrointestinal system (Vela et al., 2015). It is signaling may also exert an impact on synaptic plasticity in the peripheral nervous system, particularly in the ENS. Zinc transporter 3 (ZnT3) has been found to be widely distributed in the ENS of both human and pigs (Gonkowski et al., 2009; Wojtkiewicz et al., 2012, 2017a,b), where it plays a crucial role in transporting zinc ions from the cytoplasm into synaptic vesicles. Wojtkiewicz et al. discovered that enteric neurons exhibit varying levels of co-localized ZnT3 alongside other key molecules, including vesicular acetylcholine transporter (vAChT), NOS, and vasoactive intestinal peptide (VIP). The degree of co-localization varies depending on the specific type of enteric plexus involved, implying that both ZnT3 and zinc may play a role in regulating multiple aspects of gut function (Wojtkiewicz et al., 2012). For instance, the majority of myenteric neurons expressing ZnT3 also exhibited immunoreactivity to vAChT and/or nNOS, which are the main excitatory and inhibitory neurons, respectively. This suggests that ZnT3 and Zn may exert a modulatory effect on the activity of both types of neurons, thereby facilitating gut smooth muscle contraction and relaxation coordination. The neurons within the submucosal plexus provide innervation to both blood vessels and secretory functions, while the presence of ZnT3 in the ENS of pig esophagus suggests a potential role for zinc and ZnT3 in sensory and pain signal transduction (Wojtkiewicz et al., 2017b). However, further investigations are necessary to clarify the precise physiological roles of ZnT3 and synaptic vesicles in the ENS across multiple species, enhancing our understanding of the mechanisms linking zinc with ASD.

## Conclusions and future prospects

In this review, we primarily summarize the potential role and underlying mechanisms of the ENS in the pathogenesis of comorbid gastrointestinal disorders observed in individuals with autism, focusing on genetic aspects. Additionally, we also present a brief overview of three environmental factors: prenatal influences, gut microbiota, and zinc, which have been implicated to varying extents in autism. Although some existing studies investigating the correlation between the ENS and ASD, there remains a dearth of substantial empirical evidence directly substantiating the link between ENS and ASD. Further investigations are warranted to enhance the existing evidence base. The impact of autism-associated mutations on the ENS presents opportunities for employing extracerebral functional assays and designing preclinical therapies in animal models, thereby enhancing the translational potential of the field involving both the peripheral and central nervous system.

## Author contributions

XW and ZL conceptualized the review. XW, RT, and ZL wrote and developed the initial drafts of the manuscript. XW developed and formatted the table. ZL organized the figure and wrote and developed advanced drafts of the manuscript with feedback from YZ and JL. All authors contributed to the article and approved the submitted version.

## Funding

This work was supported by funding from Shenzhen Science and Technology Projects (grant numbers: JCYJ20200109150700942 and JCYJ20210324140408023), as well as Guangdong Basic and Applied Basic Research Foundation (grant number: 2021A1515110787).

## Conflict of interest

The authors declare that the research was conducted in the absence of any commercial or financial relationships that could be construed as a potential conflict of interest.

## Publisher’s note

All claims expressed in this article are solely those of the authors and do not necessarily represent those of their affiliated organizations, or those of the publisher, the editors and the reviewers. Any product that may be evaluated in this article, or claim that may be made by its manufacturer, is not guaranteed or endorsed by the publisher.

## Figures and Tables

**Table:** 

ASD	Autism spectrum disorder
ChAt	Choline acetyltransferase
*CHD8*	Chromodomain helicase DNA-binding protein 8
CNS	Central nervous system
DT	Digestive tract
EECs	Enteroendocrine cells
EGC	Enteric glia cell
eGFP	Enhanced green fluorescent protein
ENS	Enteric nervous system
FOXP1	Forkhead box protein P1
GI	Gastrointestinal
HGF	Hepatocyte growth factor
KO	Knock out
Met	A human proto-oncogene
MIA	Maternal immune activation
MP	Myenteric plexus
*Nlgn3*	Neuroligin-3
nNOS	Neuronal nitric oxide synthase
PMS	Phelan-McDermid syndrome
PTHS	Pitt–Hopkins syndrome
5-HT	Serotonin
SCFAs	Short-chain fatty acids
SERT	Serotonin reuptake transporter
SMP	Submucous plexus
TCF4	Transcription factor 4
vAChT	Vesicular acetylcholine transporter
VIP	Vasoactive intestinal peptide
ZnT3	Zinc transporter 3
